# 全自动QuEChERS前处理仪结合气相色谱-四极杆-飞行时间质谱法测定橄榄油中222种农药残留

**DOI:** 10.3724/SP.J.1123.2023.09010

**Published:** 2024-04-08

**Authors:** Yan LIANG, Chunni LEI, Bo WANG, Huan ZHANG, Xinchao WANG, Xiaoping ZHOU, Zhenzhen QI, Mengchen ZHU

**Affiliations:** 1.甘肃农业大学食品科学与工程学院, 甘肃 兰州 730070; 1. College of Food Science and Engineering, Gansu Agricultural University, Lanzhou 730070, China; 2.兰州海关技术中心, 甘肃 兰州 730010; 2. Technology Center of Lanzhou Customs, Lanzhou 730010, China; 3.西北师范大学地理与环境科学学院, 甘肃 兰州 730070; 3. College of Geography and Environmental Science, Northwest Normal University, Lanzhou 730070, China

**Keywords:** 全自动QuEChERS前处理仪, 气相色谱-四极杆-飞行时间质谱, 农药残留, 橄榄油, fully automatic QuEChERS pre-treatment instrument, gas chromatography-quadrupole-time-of-flight mass spectrometry (GC-QTOF-MS), pesticide residues, olive oil

## Abstract

基于QuEChERS方法,建立了全自动QuEChERS前处理仪结合气相色谱-四极杆-飞行时间质谱法(GC-QTOF-MS)快速测定橄榄油中222种农药残留的分析方法,并考察了提取溶剂酸化程度、正己烷体积、振荡时间、离心温度、净化剂对222种农药残留测定的影响。首先选择响应较好且无明显干扰的离子用于定量及定性,再通过对全自动QuEChERS前处理仪的参数设置开发了净化流程,用含2%甲酸的乙腈溶液提取,上清液在含有400 mg *N*-丙基乙二胺(PSA)、400 mg十八烷基硅烷键合硅胶(C18)和1 200 mg无水硫酸镁的离心管中净化离心,净化液经氮气吹干,用乙酸乙酯定容后供仪器分析,最后采用基质标准溶液进行定量。从基质效应、线性范围、检出限、定量限、精密度及正确度等方面对定量分析方法进行验证。结果表明:222种农药中86.04%的农药线性范围为0.02~2.00 μg/mL, 10.81%农药的线性范围为0.10~2.00 μg/mL, 3.15%农药的线性范围为0.20~2.00 μg/mL,相关系数(*R*^2^)均大于0.99,222种农药在各自线性范围内线性关系良好;全部被测农药的检出限范围为0.002~0.050 mg/kg,定量限范围为0.007~0.167 mg/kg,其中有170种农药定量限可以达到0.007 mg/kg, 21种农药定量限可以达到0.017 mg/kg,占总数量的86.04%;在0.2、0.5和0.8 mg/kg 3个添加水平下,平均回收率为70%~120%的农药占全部被测农药的79.58%,平均回收率为80%~110%的农药占全部被测农药的65.92%,相对标准偏差(RSD, *n*=6)<10%的农药占全部被测农药的93.54%, RSD(*n*=6)<20%的农药占全部被测农药的98.35%。应用该方法对14份市售橄榄油样品进行了检测,共检出7种农药,其含量范围为0.0044~0.0490 mg/kg。该方法操作简单、快速、灵敏度高,且自动化程度较强,前处理过程无需人员值守,减轻劳动力的同时减少了人员误差,能够满足橄榄油中多种农药残留检测的需求,也可以为其他油类农药残留检测及复杂基质的自动化前处理提供参考。

## Abstract (en)

Pesticide residues may be present in olive oil because pesticides are applied to olive trees during their cultivation and growth for pest prevention and some of these pesticides are not easily degraded. Studies on pesticide residues in olive oil have mainly focused on the detection of single types of pesticide residues, and reports on the simultaneous detection of multiple pesticide residues are limited. At present, hundreds of pesticides with different polarities and chemical properties are used in practice.

In this study, an analytical method based on fully automatic QuEChERS pretreatment instrument coupled with gas chromatography-quadrupole time-of-flight mass spectrometry (GC-QTOF-MS) was established for the rapid determination of 222 pesticide residues in olive oil. The effects of acetonitrile acidification concentration, *n*-hexane volume, oscillation time, centrifugation temperature, and purification agent on the determination of the 222 pesticide residues were investigated. First, ions with good responses and no obvious interference were selected for quantification and characterization. The purification process was then developed by setting the parameters of the fully automatic QuEChERS pretreatment instrument to optimal values. The sample was extracted with acetonitrile containing 2% formic acid, and the supernatant was purified by centrifugation in a centrifuge tube containing 400 mg *N*-propylethylenediamine (PSA), 400 mg octadecylsilane-bonded silica gel (C18), and 1200 mg anhydrous magnesium sulfate. The purified solution was blown dry with nitrogen and then fixed with ethyl acetate for instrumental analysis. Finally, a matrix standard solution was used for quantification. The method was validated in terms of matrix effects, linear ranges, limits of detection (LODs) and quantification (LOQs), accuracies, and precisions. The results showed that 86.04% of the 222 pesticides had linear ranges of 0.02-2.00 μg/mL, 10.81% had linear ranges of 0.10-2.00 μg/mL, and 3.15% had linear ranges of 0.20-2.00 μg/mL. The pesticide residues showed good relationships within their respective linear ranges, and the correlation coefficients (*R*^2^) were greater than 0.99. The LODs of all tested pesticides ranged from 0.002 to 0.050 mg/kg, and their LOQs ranged from 0.007 to 0.167 mg/kg. Among the 222 pesticides determined, 170 pesticides had LOQs of 0.007 mg/kg while 21 pesticides had LOQs of 0.017 mg/kg. At the three spiked levels of 0.2, 0.5, and 0.8 mg/kg, 79.58% of all tested pesticides had average recoveries of 70%-120% while 65.92% had average recoveries of 80%-110%. In addition, 93.54% of all tested pesticides had relative standard deviations (RSDs, *n*=6)<10% while 98.35% had RSDs (*n*=6)<20%. The method was applied to 14 commercially available olive oil samples, and seven pesticides were detected in the range of 0.0044-0.0490 mg/kg. The residues of fenbuconazole, chlorpyrifos, and methoprene did not exceed the maximum limits stated in GB 2763-2021. The maximum residual limits of molinate, monolinuron, benalaxyl, and thiobencarb have not been established. The method utilizes the high mass resolution capability of TOF-MS, which can improve the detection throughput while ensuring good sensitivity. In addition, high-resolution and accurate mass measurements render the screening results more reliable, which is necessary for the high-throughput detection of pesticide residues. The use of a fully automatic QuEChERS instrument in the pretreatment step reduces personnel errors and labor costs, especially when a large number of samples must be processed, thereby offering significant advantages over other approaches. Moreover, the method is simple, rapid, sensitive, highly automatable, accurate, and precise. Thus, it meets requirements for the high-throughput detection of pesticide residues in olive oil and provides a reference for the development of detection methods for pesticide residues in other types of oils as well as the automatic pretreatment of complex matrices.

2008-2020年,我国橄榄油的消费量从1.2万吨增至5.3万吨,是世界公认的橄榄油消费大国,而国内橄榄油产量完全满足不了消费需求,2019-2020年自给率仅达到3.34%,巨大的橄榄油消费缺口只能通过进口来解决^[1]^。橄榄油是由新鲜油橄榄果实通过物理压榨法冷榨而成,不经加热及其他化学手段处理,保留了最天然的营养成分,因其具有极高的营养价值以及抗氧化、预防癌症、美容、烹饪等功能,被誉为“液体黄金”和“植物油皇后”,是迄今为止最适合人体营养的油脂^[2]^。国际橄榄理事会(IOC)将橄榄油分为初榨橄榄油、精炼橄榄油和油橄榄果渣油,品质依次递减,初榨橄榄油又分为特级初榨橄榄油、中级初榨橄榄油和初榨油橄榄油^[3]^。由于油橄榄在种植及生长过程中使用农药来达到预防虫害的目的,且部分农药不易降解,因此橄榄油中可能存在农药残留,其潜在风险不容忽视。

我国是橄榄油主要进口国,2017年中华人民共和国商业部公布:在市面上所抽检的进口欧盟橄榄油中,约53%的橄榄油含一种或多种农药残留^[4]^。目前国内外学者关于植物油中农药残留的检测方法已有报道,如蔡燕斌等^[5]^测定了食用植物油中7种农药残留;Iosif等^[6]^测定了橄榄油中39种农药残留等,其中橄榄油农药残留的研究主要集中于一种或一类农药残留的检测,针对数百种不同极性、不同化学性质的多种农药残留的检测报道较少。实现农业长远健康发展必须不断加大对农产品质量的检测检验工作,农药残留是农产品质量把控的重要内容,定期、及时的开展农药残留监测十分必要,通过建立橄榄油中快速、高效并同时实现高通量筛查的多农药残留检测方法,以有效保证橄榄油质量安全、保护进出口公司利益,为提高口岸风险监测效率、加强监管力度提供技术支撑。

橄榄油或植物油样品中农药残留的前处理方法主要有固相萃取法(SPE)^[7]^、凝胶渗透色谱法(GPC)^[8]^、分散-液液微萃取法(DLLME)^[9]^、QuEChERS法^[10]^等。QuEChERS法是由Anastassiades等^[11]^于2003年首次提出,该技术分析速率快、使用溶剂量少、污染小、操作简单,已被广泛应用于农药残留分析。全自动QuEChERS前处理仪是自动程序控制的多功能样品制备工作站,它建立在六轴机械手平台上,集合样品管理、液体处理、开关盖、振荡提取、加盐模块、离心分离等六大模块,使QuEChERS方法样品制备过程无人化,减轻劳动力的同时减少了人员误差,极大地提高了样品的前处理效率。扈斌等^[12]^利用自动QuEChERS法检测香叶中212种农药残留,蒋康丽等^[13]^利用自动QuEChERS法检测花生中297种农药残留。以上研究结果表明:全自动QuEChERS前处理仪应用于农药残留检测,具有简便、快速、灵敏度高且自动化程度高等优势。然而运用全自动QuEChERS前处理仪检测橄榄油中农药多残留的分析鲜见报道。

农药残留分析常用技术有GC^[14]^、GC-MS^[15]^、GC-MS/MS^[16]^、LC、LC-MS、LC-MS/MS^[17]^等。色谱与质谱联用具有极大优势,可充分发挥高分离及高鉴别能力,提供高效、快速的微量组分分析和结构鉴定能力。气相色谱-四极杆-飞行时间质谱法(GC-QTOF-MS)具有高质量分辨率、高扫描速率等特点,在农药残留的非靶向筛查和定量检测方面具有极其广阔的应用前景,此外高分辨率精确质量测量使筛选结果更加可靠,其实验数据可进行重复分析,极大地提高了工作效率,适合农药残留的高通量检测。目前高分辨飞行时间质谱技术已应用于水果、蔬菜、谷物等样品中的农药残留分析^[18⇓-20]^,本文基于复杂基质背景下的高通量农药筛查以及自动化QuEChERS技术所带来的高度自动化等特点,针对数百种不同极性、不同化学性质的多农药残留分析展开研究,建立了一种准确、便捷且自动化程度高的橄榄油中多农药残留检测技术,以期为橄榄油中农药残留的监测以及复杂基质的自动化前处理发展提供参考。

## 1 实验部分

### 1.1 仪器、试剂与材料

GC-QTOF-MS 7890B-7200气相色谱-四极杆-飞行时间质谱仪(美国Agilent公司);全自动QuEChERS前处理仪,15 mL、50 mL QuEChERS离心管(睿科集团(厦门)股份有限公司); BSA822电子天平(赛多利斯科学仪器(北京)有限公司)。

222种农药标准品,均溶于乙酸乙酯,质量浓度为100 μg/mL(天津阿尔塔科技有限公司);正己烷(分析纯,国药集团化学试剂有限公司);乙腈、乙酸乙酯(分析纯,天津市富宇精细化工有限公司);甲酸、无水硫酸镁(分析纯,天津市百世化工有限公司);十八烷基硅烷键合硅胶(C18)粉末、*N*-丙基乙二胺(PSA)粉末(美国Agilent公司)。橄榄油样品均为市售特级初榨橄榄油(进口、国产)。

### 1.2 样品前处理

称取2.0 g橄榄油试样,置于50 mL离心管中,放入全自动QuEChERS前处理仪,通过参数设置加入10 mL 2%甲酸乙腈溶液提取,后经5 Hz频率垂直振荡2 min,并在4 ℃下以3500 r/min冷冻离心5 min。取7 mL上清液加入含有400 mg PSA、400 mg C18、1200 mg无水硫酸镁的离心管中净化离心,净化时设置垂直振荡频率5 Hz、时长2 min,离心温度4 ℃、转速4000 r/min、时长5 min。待净化、离心等步骤处理完成后,取4 mL净化液氮气吹干,用乙酸乙酯定容至0.8 mL后转移至进样瓶,进行GC-QTOF-MS测定分析。

### 1.3 标准溶液的配制

将222种农药标准品用乙酸乙酯配制得到质量浓度为10 μg/mL的农药混合标准储备溶液。混合标准储备溶液用经1.2节方法处理的空白橄榄油基质提取液逐级稀释,配制成系列质量浓度的基质混合标准溶液。

### 1.4 GC-QTOF-MS仪器条件

#### 1.4.1 色谱条件

色谱柱:HP-5MS UI柱(30 m×250 μm×0. 25 μm);进样口温度:280 ℃;进样模式:不分流进样;载气:氦气,纯度≥99.999%;载气流速:1 mL/min;升温程序:初始温度50 ℃,保持2 min,以40 ℃/min升温至120 ℃,再以3 ℃/min升温至255 ℃,最后以10 ℃/min升温至300 ℃,保持6 min;进样量:1 μL。

#### 1.4.2 质谱条件

离子化模式:电子轰击(EI)源;电子能量:70 eV;离子源温度:230 ℃;监测模式:全扫描模式;扫描范围:*m/z* 35~500;扫描速率:5 spectra/s;四极杆温度:150 ℃;碰撞气:高纯氮气(99.999%)。

### 1.5 数据处理

数据采集通过Agilent Mass Hunter Acquisition 7200软件完成,检测结果的数据处理通过Agilent Mass Hunter Qualitative Analysis B.07.00软件完成,数据分析通过Agilent Mass Hunter TOF定量分析软件分析。每种农药选择响应较好且无明显干扰的离子用于定量及定性,并参照GB 23200.8-2016 《食品安全国家标准 水果和蔬菜中500种农药及相关化学品残留量的测定 气相色谱-质谱法》^[21]^选定一个定量离子及两个定性离子(见表1)。

**表 1:** 橄榄油中222种农药的保留时间、定量离子、定性离子、线性范围、检出限和定量限

Pesticide			t_R_/min			Quantitative ion (m/z)			Qualitative ions (m/z)			Linear range/ (μg/mL)		LOD/ (mg/kg)		LOQ/ (mg/kg)	
Ethiolate (硫草敌)			7.664			100.0892			118.0609, 161.0781			0.02-2		0.002		0.007	
Methamidophos (甲胺磷)			8.085			94.0009			95.0077, 140.9910			0.2-2		0.050		0.167	
Dichlorvos (敌敌畏)			8.407			109.0083			184.9746, 219.9314			0.02-2		0.002		0.007	
Dichlorobenzonitrile (敌草腈)			10.367			170.9696			172.9625, 135.9867			0.02-2		0.002		0.007	
Biphenyl (联苯)			11.121			154.1009			153.0796, 152.0691			0.1-2		0.010		0.033	
Mevinphos (速灭磷)			12.341			127.0167			192.0068, 164.0132			0.02-2		0.002		0.007	
Acephate (乙酰甲胺磷)			12.401			136.0064			93.9982, 42.0310			0.2-2		0.050		0.167	
Etridiazole (土菌灵)			12.975			210.9427			182.9124, 123.9262			0.02-2		0.002		0.007	
Methacrifos (虫螨畏)			14.345			124.9783			207.9900, 240.0058			0.02-2		0.002		0.007	
Chloroneb (氯苯甲醚)			14.495			190.9667			192.9600, 205.9843			0.02-2		0.002		0.007	
Molinate (禾草敌)			15.165			126.0920			187.0895, 98.0902			0.02-2		0.002		0.007	
Isoprocarb (异丙威)			15.341			121.0716			136.0862, 103.0472			0.1-2		0.020		0.067	
Omethoate (氧乐果)			16.920			155.9903			110.0052, 78.9883			0.2-2		0.050		0.167	
Tecnazene (四氯硝基苯)			17.232			260.8624			202.8758, 214.8703			0.02-2		0.002		0.007	
Thionazin (虫线磷)			17.371			142.9932			191.9619, 219.9912			0.02-2		0.002		0.007	
Fenobucarb (仲丁威)			17.437			121.0760			150.1006, 107.0426			0.2-2		0.050		0.167	
Propoxur (残杀威)			17.564			110.0438			152.0756, 111.0321			0.1-2		0.020		0.067	
Diphenylamine (二苯胺)			17.736			169.0927			167.0702, 168.0827			0.02-2		0.002		0.007	
Cycloate (环草敌)			18.147			154.1340			186.0826, 215.1187			0.02-2		0.002		0.007	
Ethoprophos (灭线磷)			18.197			157.9584			199.9963, 242.0395			0.02-2		0.002		0.007	
Chlorpropham (氯苯胺灵)			18.758			213.0427			170.9985, 152.9868			0.02-2		0.005		0.017	
Naled (二溴磷)			18.877			78.9884			108.9983, 112.9463			0.2-2		0.050		0.167	
Atrazine-desethyl (脱乙基莠去津)			19.183			172.0505			187.0554, 145.0109			0.02-2		0.002		0.007	
Ethalfluralin (乙丁烯氟灵)			19.196			276.0444			316.0723, 292.0355			0.02-2		0.002		0.007	
Dicrotofos (百治磷)			19.461			127.0112			94.9817, 108.9966			0.02-2		0.005		0.017	
Benfluralin (乙丁氟灵)			19.909			292.0515			264.0108, 276.0418			0.02-2		0.002		0.007	
Monocrotophos (久效磷)			19.916			127.0099			97.0445, 108.9964			0.1-2		0.020		0.067	
Sulfotep (治螟磷)			19.936			322.0146			201.9799, 237.9182			0.02-2		0.002		0.007	
Phorate (甲拌磷)			20.052			259.9954			121.0357, 230.9612			0.02-2		0.002		0.007	
α-Hexachlorocyclohexane (α-六六六)			20.112			218.9042			182.9330, 220.8959			0.02-2		0.002		0.007	
Hexachlorobenzene (六氯苯)			20.566			283.8078			285.8024, 281.8039			0.02-2		0.002		0.007	
Dicloran (氯硝胺)			20.914			175.9603			205.9528, 123.9870			0.02-2		0.005		0.017	
Dimethoate (乐果)			21.097			87.0095			124.9745, 228.9845			0.02-2		0.005		0.017	
Atratone (阿特拉通)			21.256			196.1111			211.1316, 197.1077			0.02-2		0.002		0.007	
Carbofuran (克百威)			21.691			164.0770			131.0396, 149.0518			0.02-2		0.002		0.007	
Simazine (西玛津)			21.877			201.0662			186.0428, 173.0353			0.02-2		0.002		0.007	
Atrazine (莠去津)			21.880			200.0647			215.0820, 173.0357			0.02-2		0.002		0.007	
Monolinuron (绿谷隆)			21.908			61.0492			126.0029, 214.0377			0.1-2		0.020		0.067	
Clomazone (异噁草酮)			21.910			204.0997			138.0002, 205.0914			0.02-2		0.002		0.007	
β-Hexachlorocyclohexane (β-六六六)			22.109			218.9013			216.9025, 180.9314			0.02-2		0.002		0.007	
γ-Hexachlorocyclohexane (γ-六六六)			22.109			182.9336			218.9054, 253.8657			0.02-2		0.002		0.007	
Propazine (扑灭津)			22.175			214.0791			229.0980, 172.0329			0.02-2		0.002		0.007	
Pentachloronitrobenzene (五氯硝基苯)			22.457			294.8278			236.8424, 248.8362			0.02-2		0.002		0.007	
Terbufos (特丁硫磷)			22.686			230.9712			153.0030, 288.0232			0.02-2		0.002		0.007	
Terbuthylazine (特丁津)			22.756			214.0771			229.0932, 173.0370			0.02-2		0.002		0.007	
Fonofos (地虫硫磷)			22.759			246.0186			137.0167, 173.9606			0.02-2		0.002		0.007	
Propetamphos (胺丙畏)			22.961			138.0151			193.9762, 236.0145			0.02-2		0.002		0.007	
Pronamide (炔苯酰草胺)			22.965			172.9631			255.0082, 239.9837			0.02-2		0.002		0.007	
Profluralin (环丙氟灵)			23.167			318.0563			302.0540, 347.0856			0.02-2		0.002		0.007	
Pyrimethanil (嘧霉胺)			23.197			198.1227			199.1151, 200.1024			0.02-2		0.002		0.007	
Diazinon (二嗪磷)			23.685			304.0832			179.1140, 137.0686			0.02-2		0.002		0.007	
δ-Hexachlorocyclohexane (δ-六六六)			23.711			218.8959			216.8989, 180.9252			0.02-2		0.002		0.007	
Paraoxon-methyl (甲基对氧磷)			23.778			230.0102			247.0119, 200.0130			0.1-2		0.020		0.067	
Triallate (野麦畏)			24.242			268.0215			270.0159, 142.9132			0.02-2		0.002		0.007	
Isazofos (氯唑磷)			24.381			161.0293			256.9648, 284.9919			0.02-2		0.002		0.007	
Etrimfos (乙嘧硫磷)			24.561			292.0529			181.0911, 277.0242			0.02-2		0.002		0.007	
Iprobenfos (异稻瘟净)			24.750			203.9990			246.0325, 288.0748			0.02-2		0.002		0.007	
Tebupirimfos (丁基嘧啶磷)			24.863			318.1006			261.0352, 234.0133			0.02-2		0.002		0.007	
Pentachloroaniline (五氯苯胺)			24.919			264.8560			262.8546, 229.8748			0.02-2		0.002		0.007	
Formothion (安硫磷)			24.995			169.9500			223.9988, 256.9761			0.1-2		0.010		0.033	
Pirimicarb (抗蚜威)			25.218			166.1011			238.1300, 138.0683			0.02-2		0.002		0.007	
Desmetryn (敌草净)			25.569			213.0994			198.0735, 171.0488			0.02-2		0.005		0.017	
Dichlofenthion (除线磷)			25.768			278.9934			222.9357, 250.9569			0.02-2		0.002		0.007	
Propanil (敌稗)			25.818			160.9772			216.9907, 162.9712			0.02-2		0.002		0.007	
Metribuzin (嗪草酮)			25.878			198.0642			199.0560, 144.0356			0.1-2		0.010		0.033	
Phosphamidon (磷胺)			25.904			264.0847			138.0818, 226.9717			0.1-2		0.010		0.033	
Acetochlor (乙草胺)			26.239			146.0888			162.0820, 223.0629			0.02-2		0.005		0.017	
Parathion-methyl (甲基对硫磷)			26.279			262.9886			233.0082, 245.9810			0.02-2		0.002		0.007	
Chlorpyrifos-methyl (甲基毒死蜱)			26.283			285.9319			287.9240, 196.9072			0.02-2		0.005		0.017	
Vinclozolin (乙烯菌核利)			26.346			284.9771			211.9911, 197.9749			0.02-2		0.002		0.007	
Tolclofos-methyl (甲基立枯磷)			26.515			265.0004			266.9819, 249.9527			0.02-2		0.002		0.007	
Malaoxon (马拉氧磷)			26.704			127.0199			267.9982, 194.9738			0.1-2		0.010		0.033	
Alachlor (甲草胺)			26.813			188.1010			237.0754, 269.0989			0.02-2		0.002		0.007	
Ronnel (皮蝇磷)			27.172			284.9383			286.9299, 124.9791			0.02-2		0.002		0.007	
Metalaxyl (甲霜灵)			27.208			206.1056			249.1186, 234.0966			0.02-2		0.002		0.007	
Ametryn (莠灭净)			27.221			227.1147			212.0883, 185.0617			0.02-2		0.002		0.007	
Prometryn (扑草净)			27.221			241.1373			184.0705, 226.1089			0.02-2		0.002		0.007	
Paraoxon (对氧磷)			27.228			275.0425			219.9897, 247.0123			0.1-2		0.020		0.067	
Terbutryn (特丁净)			27.951			226.1065			241.1234, 185.0702			0.02-2		0.002		0.007	
Fenitrothion (杀螟硫磷)			28.057			277.0019			260.0004, 247.0253			0.02-2		0.002		0.007	
Pirimiphos-methyl (甲基嘧啶磷)			28.339			290.0641			276.0468, 305.0817			0.02-2		0.002		0.007	
Ethofumesate (乙氧呋草黄)			28.343			207.1015			161.0605, 286.0749			0.02-2		0.005		0.017	
Bromacil (除草定)			28.353			204.9531			206.9509, 230.9601			0.02-2		0.002		0.007	
Aldrin (艾氏剂)			28.509			262.8441			264.8383, 292.9078			0.02-2		0.002		0.007	
Phorate sulfoxide (甲拌磷亚砜)			28.522			276.8945			142.9300, 198.9867			0.1-2		0.020		0.067	
Thiobencarb (禾草丹)			28.655			100.0792			125.0116, 257.0521			0.02-2		0.002		0.007	
Dipropetryn (异丙净)			28.827			255.1433			240.1147, 222.1578			0.02-2		0.002		0.007	
Malathion (马拉硫磷)			28.907			173.0771			157.9537, 142.9852			0.02-2		0.005		0.017	
Phorate sulfone (甲拌磷砜)			29.000			198.9963			170.9637, 214.9855			0.02-2		0.002		0.007	
Metolachlor (异丙甲草胺)			29.030			238.0921			162.1263, 240.0823			0.02-2		0.002		0.007	
Fenthion (倍硫磷)			29.252			278.0226			169.0053, 153.0263			0.02-2		0.002		0.007	
Chlorpyrifos (毒死蜱)			29.388			313.9399			257.8800, 285.9072			0.02-2		0.002		0.007	
Parathion (对硫磷)			29.431			291.0141			185.9818, 234.9534			0.02-2		0.002		0.007	
Triadimefon (三唑酮)			29.600			208.0170			210.0096, 181.0042			0.02-2		0.002		0.007	
Trichloronat (毒壤磷)			30.061			296.9569			268.9284, 195.9114			0.02-2		0.002		0.007	
Tetraconazole (四氟醚唑)			30.124			336.0521			338.0360, 170.9659			0.02-2		0.002		0.007	
Isocarbophos (水胺硫磷)			30.131			135.9955			229.9885, 289.0336			0.1-2		0.010		0.033	
Isofenphos-oxon (氧异柳磷)			30.214			229.0271			200.9983, 314.0973			0.02-2		0.002		0.007	
Bromophos (溴硫磷)			30.314			330.8732			328.8720, 212.8542			0.02-2		0.002		0.007	
Fosthiazate (噻唑磷)			30.493			194.9978			226.9682, 283.0267			0.1-2		0.020		0.067	
Cyprodinil (嘧菌环胺)			30.864			224.1262			225.1251, 210.0900			0.02-2		0.002		0.007	
Pirimiphos-ethyl (嘧啶磷)			30.980			333.1124			318.0908, 304.0730			0.02-2		0.002		0.007	
Terbufos sulfone (特丁硫磷砜)			31.030			230.9698			288.0501, 185.9850			0.02-2		0.002		0.007	
Isofenphos-methyl (甲基异柳磷)			31.037			199.0125			230.9753, 241.0477			0.02-2		0.002		0.007	
Pendimethalin (二甲戊灵)			31.339			252.0933			220.0907, 162.0677			0.02-2		0.002		0.007	
Penconazole (戊菌唑)			31.392			248.0891			250.0777, 160.9680			0.02-2		0.002		0.007	
Mephosfolan (地胺磷)			31.807			196.0078			226.9698, 167.9790			0.02-2		0.005		0.017	
Phosfolan (硫环磷)			31.807			196.0056			139.9479, 167.9762			0.02-2		0.005		0.017	
Chlorfenvinphos (毒虫畏)			31.976			322.9819			266.9282, 268.9218			0.02-2		0.002		0.007	
Isofenphos (异柳磷)			32.031			213.0393			255.0684, 185.0048			0.02-2		0.002		0.007	
Quinalphos (喹硫磷)			32.049			146.0470			298.0389, 157.0727			0.02-2		0.002		0.007	
Triadimenol (三唑醇)			32.105			112.0441			168.1019, 129.9898			0.02-2		0.005		0.017	
Allethrin (烯丙菊酯)			32.231			123.1238			105.0626, 134.0626			0.02-2		0.002		0.007	
Fipronil (氟虫腈)			32.268			366.9288			368.9224, 350.9279			0.02-2		0.002		0.007	
Beflubutamid (氟丁酰草胺)			32.298			176.1074			193.0209, 221.0503			0.02-2		0.002		0.007	
Procymidone (腐霉利)			32.384			283.0095			285.0025, 255.0050			0.02-2		0.002		0.007	
trans-Chlordane (反式-氯丹)			32.483			372.8155			374.8121, 376.8029			0.02-2		0.002		0.007	
Methoprene (烯虫酯)			32.762			73.0648			191.1665, 153.0803			0.02-2		0.002		0.007	
Methidathion (杀扑磷)			32.765			145.0061			156.9427, 301.9400		0.02-2			0.002		0.007	
o,p'-DDE (o,p'-滴滴伊)			33.001			246.0098			317.9305, 176.0581			0.02-2		0.002		0.007	
Bromophos-ethyl (乙基溴硫磷)			33.041			358.8926			302.8351, 356.8924			0.02-2		0.002		0.007	
Paclobutrazol (多效唑)			33.087			236.0509			238.0410, 167.0151			0.02-2		0.002		0.007	
α-Endosulfan (α-硫丹)			33.147			240.8893			264.8366, 338.8507			0.02-2		0.002		0.007	
Fenothiocarb (苯硫威)			33.273			72.0496			160.0766, 253.1013			0.02-2		0.002		0.007	
Tetrachlorvinphose (杀虫畏)			33.562			328.9254			330.9222, 332.9077			0.02-2		0.002		0.007	
Mepanipyrim (嘧菌胺)			33.711			222.1103			223.1070, 221.0833			0.02-2		0.002		0.007	
Ditalimfos (灭菌磷)			33.820			130.0330			148.0389, 299.0278			0.02-2		0.002		0.007	
Butachior (丁草胺)			33.877			176.1016			160.1064, 188.0981			0.02-2		0.002		0.007	
Chlorfenson (杀螨酯)			34.009			301.9401			174.9608, 176.9507			0.1-2		0.010		0.033	
Napropamide (敌草胺)			34.185			271.1420			128.1027, 171.0691			0.02-2		0.002		0.007	
Hexaconazole (己唑醇)			34.285			213.9848			231.0183, 255.9863			0.02-2		0.002		0.007	
Butamifos (抑草磷)			34.308			286.0915			200.0019, 231.9714			0.02-2		0.005		0.017	
Fluorodifen (三氟硝草醚)			34.570			190.0035			328.0072, 162.0046			0.1-2		0.010		0.033	
Bromfenvinfos (溴苯烯磷)			34.583			266.9329			322.9840, 294.9512			0.02-2		0.002		0.007	
Prothiofos (丙硫磷)			34.583			308.9848			266.9392, 161.9586			0.02-2		0.002		0.007	
Imazalil (抑霉唑)			34.600			214.9888			172.9450, 296.0288			0.02-2		0.002		0.007	
Flutolanil (氟酰胺)			34.646			173.0370			281.0648, 323.1041			0.02-2		0.002		0.007	
Isoprothiolane (稻瘟灵)			34.723			290.0518			231.0042, 204.0208			0.02-2		0.002		0.007	
Dieldrin (狄氏剂)			34.726			262.8407			276.8541, 379.8423			0.02-2		0.002		0.007	
Profenofos (丙溴磷)			34.786			338.9458			373.9085, 296.8982			0.02-2		0.002		0.007	
p,p'-DDE (p,p'-滴滴伊)			34.942			317.9264			315.9269, 245.9984			0.02-2		0.002		0.007	
Fludioxonil (咯菌腈)			35.078			248.0389			127.0367, 154.0442			0.02-2		0.002		0.007	
Tribufos (脱叶磷)			35.084			201.9631			226.0507, 258.0214			0.02-2		0.002		0.007	
Pretilachlor (丙草胺)			35.084			238.0887			202.1120, 262.1641			0.02-2		0.002		0.007	
o,p'-DDD (o,p'-滴滴滴)			35.416			235.0092			237.0020, 165.0656			0.02-2		0.002		0.007	
Oxadiazon (噁草酮)			35.492			174.9555			258.0203, 302.0050			0.02-2		0.002		0.007	
Myclobutanil (腈菌唑)			35.539			179.0302			288.0941, 150.0029			0.1-2		0.010		0.033	
Oxyfluorfen (乙氧氟草醚)			35.924			252.0335			361.0175, 299.9916			0.02-2		0.002		0.007	
Endrin (异狄氏剂)			35.970			262.8402			316.9855, 344.8754			0.02-2		0.002		0.007	
Bupirimate (乙嘧酚磺酸酯)			36.047			273.0912			316.1370, 208.1364			0.02-2		0.002		0.007	
Nitrofen (除草醚)			36.156			282.9688			252.9662, 202.0076			0.02-2		0.002		0.007	
Kresoxim-methyl (醚菌酯)			36.176			116.0527			206.0747, 131.0703			0.02-2		0.002		0.007	
Cyproconazole (环丙唑醇)			36.216			222.0360			224.0290, 223.0340			0.02-2		0.002		0.007	
Isoxathion (噁唑啉)			36.285			313.0337			105.0291, 177.0167			0.1-2		0.020		0.067	
β-Endosulfan (β-硫丹)			36.594			240.8945			264.8716, 338.8511			0.02-2		0.002		0.007	
Fluazifop-butyl (吡氟禾草灵)			36.863			282.0727			383.1197, 254.0441			0.02-2		0.002		0.007	
Cyflufenamid (环氟菌胺)			36.866			91.0556			412.1057, 294.0675			0.02-2		0.002		0.007	
Chlorobenzilate (乙酯杀螨醇)			36.959			251.0241			253.0118, 152.0522			0.02-2		0.002		0.007	
Fenthion sulfoxide (倍硫磷亚砜)			37.211			278.0210			279.0198, 294.0194			0.02-2		0.002		0.007	
Fensulfothion (丰索磷)			37.214			292.0167			308.0106, 292.9980			0.02-2		0.002		0.007	
Diniconazole (烯效唑)			37.228			267.9920			269.9865, 232.0118			0.02-2		0.002		0.007	
Aclonifen (苯草醚)			37.420			264.0316			212.0507, 266.0158			0.02-2		0.002		0.007	
o,p'-DDT (o,p'-滴滴涕)			37.553			235.0120			237.0045, 165.0672			0.02-2		0.002		0.007	
Oxadixyl (噁霜灵)			37.722			163.0906			233.0769, 278.1072			0.1-2		0.010		0.033	
p,p'-DDD (p,p'-滴滴滴)			37.729			235.0081			237.0011, 199.0195			0.02-2		0.002		0.007	
Ethion (乙硫磷)			37.924			230.9695			383.9613, 198.9868			0.02-2		0.002		0.007	
Chlorthiophos (虫螨磷)			38.104			324.9752			359.9349, 296.9411			0.02-2		0.002		0.007	
Triazophos (三唑磷)			38.701			161.0492			172.0750, 256.9840			0.02-2		0.002		0.007	
Carbophenothion (三硫磷)			38.993			156.9838			341.9531, 198.9884			0.02-2		0.002		0.007	
Benalaxyl (苯霜灵)			39.152			148.1155			206.1085, 325.1462			0.02-2		0.002		0.007	
Edifenphos (敌瘟磷)			39.165			310.0146			172.9787, 201.0044			0.02-2		0.002		0.007	
Famphur (伐灭磷)			39.165			218.0307			124.9781, 216.9966			0.02-2		0.002		0.007	
Quinoxyfen (喹氧灵)			39.238			237.0510			272.0121, 306.9770			0.02-2		0.002		0.007	
Propiconazole (丙环唑)			39.507			259.0143			172.9486, 261.0098			0.02-2		0.002		0.007	
p,p'-DDT (p,p'-滴滴涕)			39.606			235.0079			237.0011, 245.9832			0.02-2		0.002		0.007	
Trifloxystrobin (肟菌酯)			40.230			116.0482			131.0682, 222.0624			0.02-2		0.002		0.007	
Hexazinone (环嗪酮)			40.290			171.0916			252.1403, 128.0729			0.02-2		0.005		0.017	
Tebuconazole (戊唑醇)			40.429			250.0604			163.0190, 252.0540			0.1-2		0.010		0.033	
Diclofop-methyl (禾草灵)			40.824			252.9747			280.9980, 342.0070			0.02-2		0.002		0.007	
Fenthion sulfone (倍硫磷砜)			41.249			309.9884			136.0236, 231.0079			0.2-2		0.050		0.167	
Piperonyl butoxide (增效醚)			41.342			176.0851			149.0510, 177.0810			0.02-2		0.002		0.007	
Epoxiconazole (氟环唑)			41.507			192.0245			183.0484, 138.0017			0.02-2		0.005		0.017	
Iprodione (异菌脲)			42.304			186.9480			244.9718, 245.9635			0.02-2		0.005		0.017	
Phosmet (亚胺硫磷)			42.393			160.0480			161.0339, 316.9728			0.02-2		0.002		0.007	
Pyridaphenthion (哒嗪硫磷)			42.466			340.0495			199.0774, 188.0462			0.02-2		0.002		0.007	
Tetramethrin (胺菊酯)			42.669			164.0711			165.0613, 135.0338			0.02-2		0.002		0.007	
Bromopropylate (溴螨酯)			42.795			340.8932			182.9389, 338.8881			0.02-2		0.002		0.007	
Phosphonothioic acid (苯硫磷)			42.873			156.9916			169.0369, 323.0179			0.02-2		0.005		0.017	
Piperophos (哌草磷)			43.093			320.1320			140.1009, 122.0916			0.02-2		0.002		0.007	
Dicofol (三氯杀螨醇)			43.103			138.9872			140.9822, 249.9779			0.02-2		0.020		0.067	
Mothoxychlor (甲氧滴滴涕)			43.130			227.1176			228.1008, 212.0698			0.02-2		0.002		0.007	
Bifenthrin (联苯菊酯)			43.213			181.1216			166.0839, 165.0741			0.02-2		0.002		0.007	
Fenpropathrin (甲氰菊酯)			43.462			265.0586			181.0619, 349.1436			0.02-2		0.002		0.007	
Fenamidone (咪唑菌酮)			43.558			268.0885			238.1106, 206.0665			0.02-2		0.005		0.017	
Etoxazole (乙螨唑)			43.604			300.1014			330.1098, 359.1463			0.02-2		0.002		0.007	
Tebufenpyrad (吡螨胺)			43.644			318.1257			333.1460, 276.0770			0.02-2		0.002		0.007	
Bifenox (甲羧除草醚)			43.797			340.9716			309.9480, 342.9655			0.1-2		0.020		0.067	
Anilofos (莎稗磷)			43.896			226.0439			183.9984, 334.0212			0.02-2		0.002		0.007	
Tetradifon (三氯杀螨砜)			44.128			226.8787			355.8862, 158.9619			0.02-2		0.002		0.007	
Phosalone (伏杀硫磷)			44.775			181.9980			366.9629, 154.0091			0.02-2		0.002		0.007	
Leptophos (溴苯磷)			44.845			376.8865			374.8858, 378.8734			0.02-2		0.002		0.007	
Pyriproxyfen (吡丙醚)			45.210			136.0821			226.0844, 185.0466			0.2-2		0.050		0.167	
Mefenacet (苯噻酰草胺)			45.354			192.0106			120.0732, 136.0140			0.02-2		0.002		0.007	
Fenarimol (氯苯嘧啶醇)			46.242			138.9904			219.0196, 330.0112			0.02-2		0.002		0.007	
Iambda-cyhalothrin (高效氯氟氰菊酯)			46.451			181.0636			197.0287, 141.0438			0.02-2		0.002		0.007	
Azinphos (益棉磷)			46.776			160.0417			132.0434, 77.0350			0.02-2		0.005		0.017	
Pyrazophos (吡菌磷)			47.005			221.0761			232.0964, 373.0626			0.02-2		0.002		0.007	
Acrinathrin (氟丙菊酯)			47.294			181.0606			289.0513, 247.0059			0.02-2		0.002		0.007	
Permethrin (氯菊酯)			48.528			183.0765			184.0712, 255.0391			0.02-2		0.002		0.007	
Pyridaben (哒螨灵)			48.747			147.1213			117.0628, 364.1121			0.02-2		0.002		0.007	
Fluquinconazole (氟喹唑)			49.009			340.0395			342.0248, 341.0252			0.02-2		0.002		0.007	
Coumaphos (蝇毒磷)			49.075			361.9983			225.9739, 363.9892			0.02-2		0.002		0.007	
Dioxathion (敌噁磷)			49.467			269.9964			197.0255, 168.9962			0.1-2		0.020		0.067	
Fenbuconazole (腈苯唑)			50.044			129.0564			198.0787, 125.0073			0.02-2		0.002		0.007	
Cyfluthrin (氟氯氰菊酯)			50.711			206.0457			199.0421, 226.0504			0.02-2		0.005		0.017	
Boscalid (啶酰菌胺)			50.900			139.9894			342.0169, 344.0113			0.02-2		0.002		0.007	
Cypermethrin (氯氰菊酯)			51.378			181.0535			152.0515, 180.0686			0.02-2		0.005		0.017	
Flucythrinate (氟氰戊菊酯)			51.500			199.0846			157.0405, 451.1299			0.02-2		0.002		0.007	
Fenvalerate (氰戊菊酯)			52.745			167.0533			225.0676, 419.1027			0.02-2		0.002		0.007	
Fluvalinate (氟胺氰菊酯)			53.259			250.0609			252.0467, 181.0552			0.02-2		0.002		0.007	
Difenoconazole (苯醚甲环唑)			53.511			323.0021			324.9986, 264.9639			0.1-2		0.020		0.067	
Deltamethrin (溴氰菊酯)			54.082			181.0551			171.9773, 173.9753			0.02-2		0.002		0.007	

## 2 结果与讨论

### 2.1 色谱条件的优化

农药残留分析中最普遍使用的两种色谱柱是弱极性HP-5毛细管柱和中等极性的HP-1701毛细管柱,其中HP-1701毛细管柱不适用于部分高沸点菊酯类及唑类农药的分析,弱极性HP-5毛细管柱在分析极性较强化合物时易出现峰拖尾现象^[22]^,因此本实验选择超高惰性的HP-5MS UI毛细管柱;同时实验考虑到少数目标物灵敏度较低,选择不分流进样;当载气流量过小时,少数目标色谱峰出现峰拖尾、峰展宽等现象,无法满足定量要求,当载气流量过大时,会影响分离度,甚至导致色谱峰重叠,不能实现目标物的有效分离^[23]^,综合考虑选择1 mL/min载气流量;升温程序在借鉴GB 23200.113-2018 《食品安全国家标准 植物源性食品中208种农药及其代谢物残留量的测定 气相色谱-质谱联用法》^[24]^色谱分析条件的基础上,延长了升温程序的时间,更有利于目标物的有效分离。加标样品中222种农药(0.5 μg/mL)的总离子流色谱图见图1。

**图 1:**
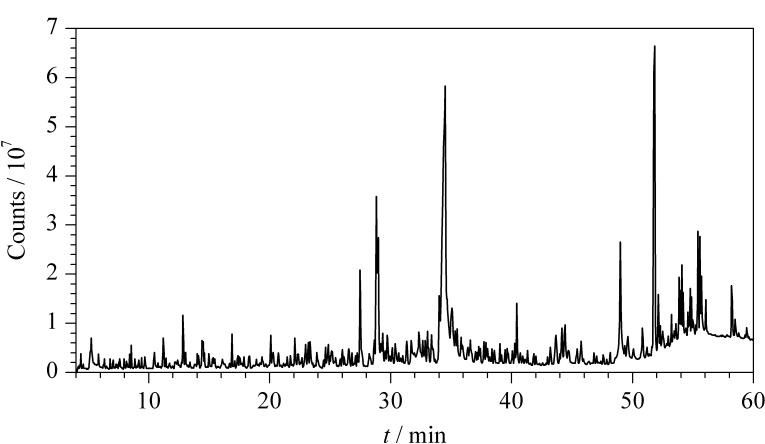
加标样品中222种农药(0.5 μg/mL)的总离子流色谱图

### 2.2 前处理条件的优化

#### 2.2.1 乙腈酸化浓度的优化

橄榄油的主要成分为油脂,油脂会给后续分析带来强烈干扰。乙腈中可溶入的油脂量极少,对绝大多数农药溶解度则较大,故采用乙腈来作为橄榄油中农药残留的提取溶剂,同时考虑到部分农药可能对酸碱度较为敏感,因此考察了含0、0.1%、0.5%、1%、2%、5%甲酸的乙腈溶液对222种农药的提取效果,结果如图2所示。222种农药的回收率在70%~120%范围的农药数量随甲酸体积分数的增大先增多后减少,说明乙腈酸化程度对农药回收率有着较大影响。在采用含2%甲酸的乙腈溶液提取时,回收率在70%~120%范围的农药数量最多,占全部被测农药的80.18%,因此选择使用含2%甲酸的乙腈溶液进行提取。

**图 2:**
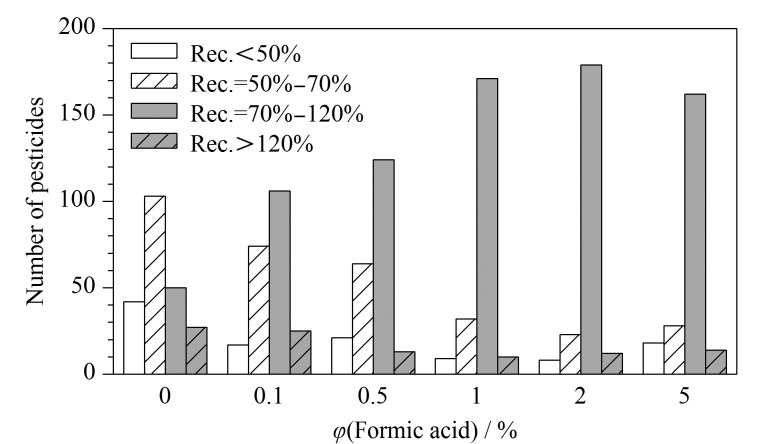
采用含不同体积分数甲酸的乙腈溶液提取时 222种农药的回收率分布

#### 2.2.2 正己烷体积的优化

橄榄油基质成分复杂,油脂干扰较大,实验预设先使用正己烷对橄榄油进行溶解,用含2%甲酸的乙腈溶液进行反提取,其中油脂溶于正己烷相,农药组分转移至乙腈相中。考察了不同正己烷体积(0、1、2、3、4 mL)对222种农药的提取效果,结果见图3。未加正己烷时,222种农药中有80.18%的农药回收率在70%~120%范围内,而加入1、2、3、4 mL正己烷时,222种农药中分别有74.77%、74.32%、74.77%和74.77%的农药回收率在70%~120%范围内。结果表明,不加正己烷时农药的回收率更高,可能是有些农药极易溶于正己烷相中,导致其回收率降低,因此选择不加正己烷,只使用含2%甲酸的乙腈溶液进行提取。

**图 3:**
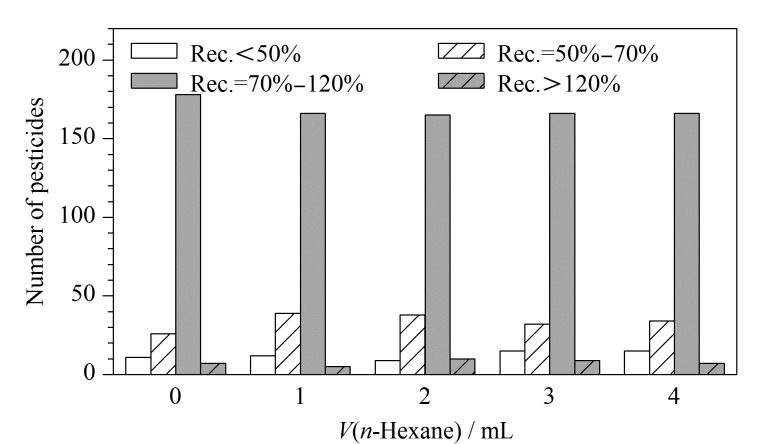
采用不同体积的正己烷提取时222种农药的回收率分布

#### 2.2.3 振荡时间的优化

全自动QuEChERS前处理仪的应用使样品处理过程无人化,减轻劳动力的同时减少了人员误差,极大地提高了样品的前处理效率和平行性。该设备具有垂直振荡和涡旋振荡两种模式,经实验室前期测试发现垂直振荡的效率更高,且在振荡频率为5 Hz时能够使基质分散均匀,可高效地进行农药残留的提取。实验对振荡频率在5 Hz时的振荡时间(1、2、3、4、5 min)进行优化,不同振荡时间下222种农药的回收率结果见图4。结果表明:振荡时间为1 min时,222种农药中有73.87%的农药回收率在70%~120%范围内;而振荡时间为2 min和3 min时,该占比分别提高至80.18%和79.28%; 3 min以后农药回收率在70%~120%范围内的数量略有下降,但基本保持不变。振荡时间为1 min时提取效率较低,可能与振荡不充分有关,导致农药未被完全提取,而振荡时间为2 min和3 min时结果基本一致,考虑到工作效率,实验选择垂直振荡2 min。

**图 4:**
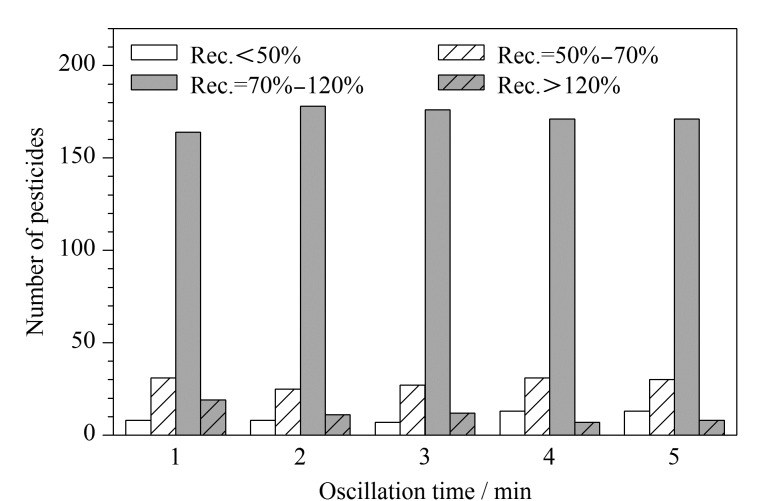
不同振荡时间提取时222种农药的回收率分布

#### 2.2.4 离心温度及净化剂的选择

根据侯靖等^[22]^在实验室前期积累的数据可知,乙腈提取液在冷冻条件下离心可以在一定程度上除去油脂类干扰物,因此在使用全自动QuEChERS前处理仪中的离心分离模块时,选择其可以设置的最低温度4 ℃作为离心温度。对于离心后的样品,考虑到依然存在少量油脂、脂肪及色素,实验参照GB 23200.113-2018^[24]^中的前处理方法,选择适用于谷物、油料和坚果净化的净化剂400 mg C18、400 mg PSA、1200 mg无水硫酸镁。

### 2.3 基质效应

基质效应是指样品基质中除分析物以外的其他成分所引起的检测信号增强或减弱^[25]^。实验对222种农药的基质效应(ME=农药在基质标准溶液中的响应/农药在溶剂标准溶液中的响应)进行了考察,ME<0.8为基质抑制效应,ME在0.8~1.2范围内为弱基质效应,ME>1.2为基质增强效应。222种农药中有6种农药表现为基质抑制效应,206种农药表现为弱基质效应,10种农药表现为基质增强效应(见附表1,详见 https://www.chrom-China.com)。为了使农药项目定量定性更加准确、可靠,实验采用空白基质配制标准溶液的方式进行定量。

### 2.4 方法学考察

#### 2.4.1 线性范围、检出限和定量限

对1.3节配制的基质混合标准溶液进行分析,以目标化合物的质量浓度为横坐标(*x*, μg/mL),峰面积为纵坐标(*y*)绘制标准曲线。如表1所示,86.04%农药的线性范围为0.02~2.00 μg/mL, 10.81%农药的线性范围为0.1~2.0 μg/mL, 3.15%农药的线性范围为0.2~2.0 μg/mL,相关系数(*R*^2^)均大于0.99,222种农药在各自线性范围内线性关系良好,满足检测要求。以3倍和10倍信噪比(*S/N*)为检出限和定量限,结果表明,222种农药的检出限为0.002~0.050 mg/kg,定量限为0.007~0.167 mg/kg,其中有170种农药定量限可以达到0.007 mg/kg, 21种农药定量限可以达到0.017 mg/kg,占总数量的86.04%; 11种农药的定量限为0.033 mg/kg, 13种农药的定量限为0.067 mg/kg, 7种农药的定量限大于0.1 mg/kg。

#### 2.4.2 正确度及精密度

在空白橄榄油试样中,分别选择0.2、0.5和0.8 mg/kg 3个水平进行加标回收试验,每个水平做6个平行,考察222种农药的正确度与精密度。结果表明:在3个添加水平下,回收率为70%~120%的农药占全部被测农药的79.58%以上,回收率为80%~110%的农药占全部被测农药的65.92%,相对标准偏差(RSD)小于10%的农药占总数的93.54%以上,RSD小于20%的农药占总数的98.35%以上(见附表1)。表明该方法准确度与精密度良好,可满足多农药残留分析的要求。

### 2.5 实际样品检测

实验对14份市售橄榄油试样进行检测,共检出7种农药,含量为0.0044~0.0490 mg/kg, 7种农药对应的提取离子色谱图见图5。

**图 5:**
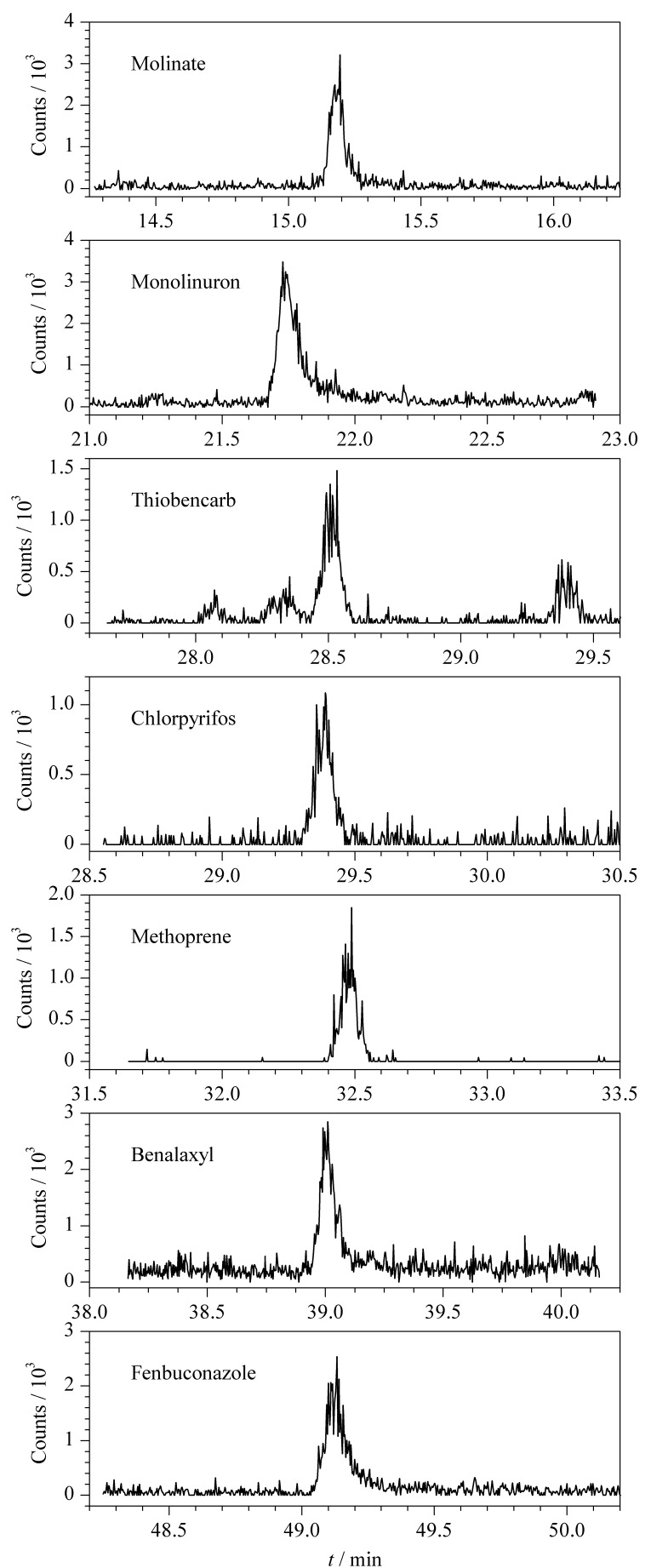
实际样品中7种农药的提取离子色谱图

其中10份橄榄油试样中均检出腈苯唑,含量为0.0148~0.0319 mg/kg;1份橄榄油试样中检出禾草敌、绿谷隆、毒死蜱、烯虫酯和腈苯唑5种农药,详细检出情况见表2。与我国现行GB 2763-2021《食品安全国家标准 食品中农药最大残留限量》^[26]^相比较,腈苯唑、毒死蜱、烯虫酯的残留量未超过其最大残留限量值,禾草敌、绿谷隆、苯霜灵、禾草丹暂未制定对应的最大残留限量值。

**表 2:** 市售橄榄油样品中农药残留的检测结果

Sample No.	Detected pesticides	Contents/(mg/kg)
1	methoprene, benalaxyl	0.0259, 0.0065
2	fenbuconazole	0.0252
3	thiobencarb	0.0044
4	fenbuconazole	0.0244
5	fenbuconazole	0.0288
6	fenbuconazole	0.0263
7	fenbuconazole	0.0293
8	fenbuconazole	0.0252
9	benalaxyl	0.0180
10	fenbuconazole, thiobencarb	0.0148, 0.0126
11	fenbuconazole	0.0240
12	fenbuconazole	0.0260
13	benalaxyl	0.0172
14	molinate, monolinuron,	0.0145, 0.0490,
	chlorpyrifos, methoprene,	0.0102, 0.0140,
	fenbuconazole	0.0319

## 3 结论

本研究将全自动QuEChERS前处理仪与气相色谱-四极杆-飞行时间质谱法相结合,建立了一种准确、便捷且自动化程度高的橄榄油多农药残留的检测方法。该方法利用飞行时间质谱的高质量分辨率特点,可以在保证灵敏度的基础上提高检测通量,此外高分辨率精确质量测量使筛选结果更加可靠,适合农药残留的高通量检测,其中前处理步骤运用全自动QuEChERS前处理仪减少了人员误差以及实验人员精力的投入,尤其是需要进行大批量样品处理时优势更加显著。该方法操作简单、快速,灵敏度高,自动化程度较强,正确度及精密度良好,满足了橄榄油中农药残留高通量检测的要求,并为其他油类农药残留检测以及复杂基质的自动化前处理发展提供了参考。
